# Myogenic Reprogramming of Bone Marrow Derived Cells in a W^41^Dmd*^mdx^* Deficient Mouse Model

**DOI:** 10.1371/journal.pone.0027500

**Published:** 2011-11-28

**Authors:** Stuart Walsh, Jens Nygren, Annica Pontén, Stefan Jovinge

**Affiliations:** 1 Lund Strategic Research Center for Stem Cell Biology and Cell Therapy, Lund University, Lund, Sweden; 2 Immunology Unit, Institution for Experimental Medical Research, Lund University, Lund, Sweden; 3 Center of Research on Welfare Health and Sport, Halmstad University, Halmstad, Sweden; 4 Department of Cardiology, Lund University Hospital, Lund, Sweden; University of Pittsburgh, United States of America

## Abstract

Lack of expression of dystrophin leads to degeneration of muscle fibers and infiltration of connective and adipose tissue. Cell transplantation therapy has been proposed as a treatment for intractable muscle degenerative disorders. Several reports have demonstrated the ability of bone-marrow derived cells (BMDC) to contribute to non-haematopoietic tissues including epithelium, heart, liver, skeletal muscle and brain following transplantation by means of fusion and reprogramming. A key issue is the extent to which fusion and reprogramming can occur *in vivo*, particularly under conditions of myogenic deterioration.

To investigate the therapeutic potential of bone marrow transplantation in monogenetic myopathy, green fluorescent protein-positive (GFP^+^) bone marrow cells were transplanted into non-irradiated c-kit receptor – deficient (W^41^) *mdx* mice. This model allows BMDC reconstitution in the absence of irradiation induced myeloablation. We provide the first report of BMDC fusion in a W^41^/Dmd*^mdx^* deficient mouse model.

In the absence of irradiation induced injury, few GFP^+^ cardiomyocytes and muscle fibres were detected 24 weeks post BMT. It was expected that the frequency of fusion in the hearts of W^41^Dmd^mdx^ mice would be similar to frequencies observed in infarcted mice [1].

Although, it is clear from this study that individual cardiomyocytes with monogenetic deficiencies can be rescued by fusion, it is as clear that in the absence of irradiation, the formation of stable and reprogrammed fusion hybrids occurs, with the current techniques, at very low levels in non-irradiated recipients.

## Introduction

Duchenne muscular dystrophy (DMD) is an X-linked recessive disease characterized by mutations in the gene encoding the membrane protein dystrophin. Lack of functional dystrophin leads to degeneration of muscle fibres and infiltration of connective and adipose tissue. Cell transplantation therapy has been proposed as a treatment for intractable muscle degenerative disorders. Several reports have demonstrated the ability of bone-marrow derived cells (BMDC) to contribute to non-haematopoietic tissues including epithelium, heart, liver, skeletal muscle and brain [1]–[10] following transplantation by means of fusion and reprogamming [11]. A key issue is the extent to which fusion and reprogramming can occur *in vivo*, particularly under conditions of myogenic deterioration.

Previously the contribution of donor BMDC has been shown to occur only in muscle regenerating after damage, and predominantly in irradiated host muscle [1], [5], [10]. Irradiation conditioning impairs regeneration from endogenous myogenic precursors and provides mitogenic conditions for donor myoblasts. Subsequently, this ablation has a major impact on the incorporation of bone marrow derived cells into the satellite cell compartment [12] and muscle fibres. However, there remains much controversy pertaining to the efficiency of transplanted BMDCs to fuse with recipient tissue. Previous reports have demonstrated that 0.2–5% of total muscle fibres are donor derived when irradiated mice are transplanted with BMDC [5], [12]–[14]. This rate of fusion remains inordinately low to derive any clinical benefits from the symptoms of DMD.

To investigate the therapeutic potential of bone marrow transplantation in DMD, green fluorescent protein-positive (GFP^+^) bone marrow cells were transplanted into non-irradiated c-kit receptor – deficient (W^41^) *mdx* mice. This model of muscular dystrophy allows BMDC reconstitution in the absence of irradiation induced myeloablation [15] ensuring that potential radiation induced fusion events do not contribute to our analyses. We provide the first report of BMDC fusion in a W^41^/Dmd*^mdx^* deficient mouse model. Although reconstitution efficiency was low, bone marrow cells contributed to reprogrammed myocytes with normal expression of dystrophin in skeletal muscle and myocardium.

## Materials and Methods

### Mice

β-actin promoter driven GFP mice [16] were used as BM donors. Haematopoietic deficient mice W^41^/W^41^ (ref 15) were back-crossed 10 generations onto the Dmd*^mdx^* strain (Jackson Laboratories). The amplification-resistant mutation assay (ARMS) was used to genotype the Dmd*^mdx^* mice [17]. Transgenic mice were all on a C57Bl/6 (Taconic) background. All mice were 8–12 weeks old at the time of transplantation. Animal experiments were performed with consent from the local ethics committee at Lund University.

### Transplantation

Whole un-fractionated bone marrow was transplanted by intravenous (tail-vein) injection into non-irradiated adult W^41^/Dmd*^mdx^* mice or sub-lethally irradiated (650 rad) W^41^/Dmd*^mdx^* controls. Individual mice received 80×10^6^ cells. Haematopoietic reconstitution was evaluated 8 weeks post transplantation and prior to sacrifice by flow cytometry.

### Anti-inflammatory treatment

Irradiated recipient W^41^/Dmd*^mdx^* mice were treated with ciprofloxacin (125 mg/l) in drinking water to prevent irradiation induced inflammation, starting from the day of irradiation [10].

### Immunohistochemistry

Chimeric mice were sacrificed by cervical dislocation. Muscle and heart tissue were perfused *in situ*, post fixed at 4°C with Stefanini solution and equilibriated in 20% sucrose, frozen and sectioned at 8–10 µm on a cryostat. Sections were stained with primary antibodies to pan-haematopoietic CD45 (Neomarkers or Abcam); cardiac troponin-T (cTNT) (Neomarkers); α-actinin (Sigma); dystrophin (Abcam); GFP (Abcam,). Secondary antibodies used were FITC donkey anti rabbit (Jackson Immunoresearch); Alexa Fluor 555 goat anti mouse (Molecular Probes); Cy5 donkey anti rat (Jackson Immunoresearch); Alexa Fluor 555 goat anti rabbit (Molecular Probes); Alexa Fluor 488 donkey anti mouse (Molecular Probes) all at pre-determined optimal concentrations. DAPI was used to visualize nuclei. Immunofluorescense images were captured using an Olympus BX51 (Olympus, Solna Sweden) fluorescence microscope and a DP70 Olympus digital camera (DP manager software Version 1.1.1.71) or a Zeisss Axiovert 200 M microscope (Carl Zeiss, Göttingen, Germany) equipped with a Zeiss ApoTome and a XB075 fluorescence lamp and a Zeiss AxioCam MRm (Zeiss Axio Vision software).

### Flow Cytometry

Blood samples were collected 8 weeks post transplantation and again prior to sacrifice after 24 and 52 weeks respectively. Peripheral blood was collected in heparinized eppendorff tubes and mixed with PBS/1% FCS. Red blood cells were sedimented and removed by Dextran 1∶1 at 37′C for 20–25 min. The cells were washed with PBS/1% FCS and further stained with pre-determined optimal concentrations of monoclonal mouse antibodies on ice for 15 minutes. After washing the cells, samples were resuspended in PBS/1% FCS and analyzed on a BD FACSCalibur. 7-amino actinomyosin (7-AAD) added prior to analysis to exclude dead cells. The following monoclonal antibodies were used for lineage readout: anti CD4-FITC, CD8-FITC, Gr-1-PE, Mac-1-PE and B220-APC (all from BD Biosciences).

### Evaluation and statistics

The frequency of GFP^+^ donor-derived non-haematopoietic cells in each tissue was determined by screening equal amounts of sections from irradiated and non-irradiated W^41^/Dmd*^mdx^* mice. Approximately 200,000 cardiomyocytes, 10,000 tibialis anterior and soleus muscle fibres per mouse were evaluated [10]. Frequency was calculated by dividing the total number of GFP^+^ cells by the number of mice and the total number of cells analyzed in each tissue. Data are presented as means ± SD.

## Results

**Figure 1:**
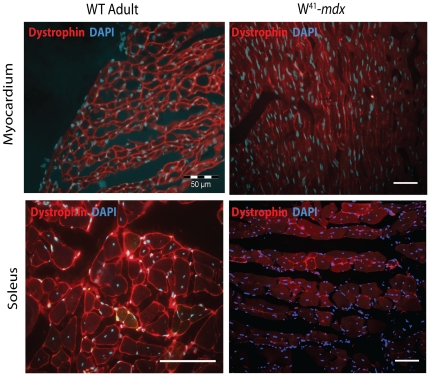
Validation of mouse model. Dystrophin phenotype in W^41^/Dmd*^mdx^* model assessed by IHC. Dystrophin localization in wild-type adult myocardium (***top left***) and soleus muscle (***bottom left***). Aberant membrane dystrophin distribution in the heart (***top right***) and soleus (***bottom right***) of deficient mice.

To investigate bone marrow as a source for cell therapy without exposing mice to irradiation, we used W^41^Dmd*^mdx^* double mutant mice. In addition to muscular dystrophy, these mice are haematopoietic deficient due to a genetically altered c-kit receptor. Dystrophin membrane localization in W^41^/Dmd*^mdx^* mice was ablated in comparison to WT expression in skeletal muscle and myocardium (**Figure 1**). Next, W^41^/Dmd*^mdx^* mice were transplanted with GFP expressing bone-marrow cells. Irradiated W^41^/Dmd*^mdx^* mice were used in control experiments. Eight weeks post transplantation W^41^Dmd*^mdx^* recipients had stable donor-derived blood reconstitution as determined by flow cytometry. GFP^+^ reconstitution in the blood of un-irradiated recipients was 11.9±9.3% and 14.4±14.2% at 24 and 52 weeks respectively (**Table 1**). Irradiated control W^41^Dmd*^mdx^* recipients had higher reconstitution at 66.4±12.6% and 82.5±10.6% at 24 and 52 weeks. Eighteen transplanted W^41^Dmd*^mdx^* were sacrificed at 24 weeks after transplantation and 20 recipients at 52 weeks to evaluate GFP expression in tibialis anterior and soleus cross sections. GFP^+^ mononucleated cells were predominantly detected in the muscle fibres of the non-irradiated group, including interstitial cells and cells adhering to or associated with muscle fibres and the lumen of vessels in myocardium (**Figure S1**). Virtually all GFP^+^ mononucleated cells were co-expressing pan-haematopoietic marker CD45.

**Table 1:** GFP^+^ bone marrow derived non-haematopoietic cells.

							GFP+ cardiomyocytes (CM)^3^		% Frequency		GFP+ muscle fibers (MF)^5^		% Frequency
				% GFP Blood Reconstitution		Numbers	Positive	Frequency	reconstitution	Numbers	Positive	Frequency	reconstitution
Treatment	n	Harvest(weeks)	Total^1^	L	M	Observed^2^	mice	GFP+ CM^4^	compensated	observed	mice	GFP+ MF	compensated
None	18	24	11.92	41.5	36.1	2	2/18	5.5×10^−07^	4.6×10^−04^	15	15/18	8.3×10^−05^	6.9×10^−02^
None	20	52	14.4	58.7	34.17	0	0/20	0	0	9	4/20	5.0×10^−05^	3.4×10^−02^
Irr	10	24	65.4	70.68	20.4	61	2/10	1.6×10^−05^	2.5×10^−03^	91	8/10	5.0×10^−04^	7.7×10^−02^
Irr	5	52	82.5	64.37	27.71	14	2/5	3.8×10^−06^	4.7×10^−04^	54	5/5	3.5×10^−04^	4.3×10^−02^

1 Mean total blood cell reconstitution of all mice in each transplant group.

2 Total number of cells detected in each tissue from all mice.

3 200,000 cardiomyocytes per mouse screened.

4 Frequency of chimeric events per tissue normalized to GFP peripheral blood reconstitution.

5 Numbers from tibialis anterior and soleus muscle were combined, 10,000 muscle fibres in total screened. Abbreviations: L, Lymphoid; M, Myeloid.

**Figure 2:**
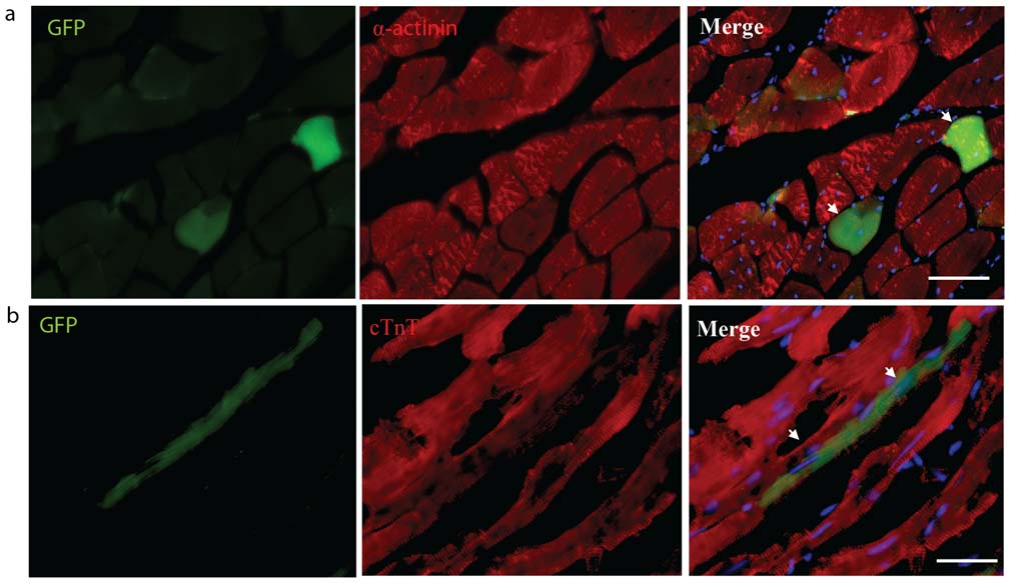
Evaluation of blood cell fusion with skeletal muscle fibres and cardiomyocytes in un-irradiated aged W^41^/Dmd*^mdx^* mice 24 weeks after bone marrow transplantation. GFP^+^ (green, left panels) bone marrow derived cells expressing lineage markers (red, middle panels) but not pan-haematopoietic CD45 (not shown). (**a**) GFP^+^ α-actinin^+^ CD45^−^ soleus muscle fibers. (**b**) GFP^+^ Cardiac Troponin-T^+^ CD45^−^ cardiomyocyte. Scale bars represent 100 µm.

Chimeric skeletal muscle fibres were defined as GFP^+^ α-actinin^+^ CD45^neg^. The distribution of GFP^+^ myofibers was inherently low in the skeletal muscles of W^41^Dmd*^mdx^* unconditioned mice with 15 and 9 fibres detected in all mice at 24 and 52 weeks respectively (**Figure 2a**). Similarly, the hearts of recipient mice were also screened for the presence of GFP^+^ cTNT^+^ CD45^neg^ cardiomyocytes, of which only two events were recorded at 24 weeks post BMT (**Figure 2b**). No chimeric myocytes were observed in the hearts of recipients 52 weeks post BMT.

**Figure 3:**
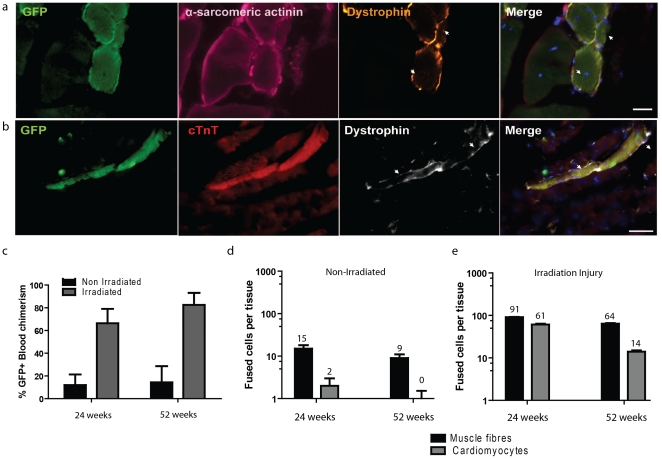
Evaluation of blood cell fusion with skeletal muscle fibres and cardiomyocytes in sub-lethally irradiated W^41^/Dmd*^mdx^* mice 24 weeks after bone marrow transplantation. GFP^+^ (green, left panels) bone marrow derived cells expressing lineage but not pan-haematopoietic CD45 (not shown) and dystrophin re-expression. (**a**) GFP^+^ α-actinin^+^ CD45^−^ soleus muscle fibres with dystrophin re-expression. (**b**) GFP^+^ Cardiac Troponin-T^+^ CD45^−^ cardiomyocyte with dystrophin re-expression. GFP^+^ recipient peripheral blood reconstitution at 24 and 52 weeks post BMT (**c**). Fused cells per tissue in non-irradiated (**d**) and irradiated recipients (**e**) at 24 and 52 weeks post BMT. Scale bars represent 50 µm. White arrows indicate dystrophin membrane expression.

In contrast to non-irradiated W^41^Dmd*^mdx^* recipients, GFP^+^ muscle fibres (**Figure 3a**); cardiomyocytes (**Figure 3b**) exhibiting characteristic morphology and lineage marker expression were discerned in the tissues of radiation conditioned W^41^Dmd*^mdx^* recipients. These donor derived cells expressed dystrophin and a total of 91 and 64 GFP^+^ skeletal muscle fibres, 61 and 14 GFP^+^ cardiomyocytes, were observed in all tissues 24 and 54 weeks after transplantation respectively (**Figure 3c–e**).

## Discussion

We sought to investigate BM contribution to myogenic lineages in a steady-state disease model of dystrophin. Several requisite conditions for BMDC conversion to the non-haematopoietic compartment in this mouse model were present at the outset of this study. Firstly, the ablation of endogenous bone marrow environment by the presence of the W^41^ mutation allowing donor derived engraftment in the absence of irradiation damage. Secondly, the exasperation for the need of regeneration induced by Dmd*^mdx^* tissue specific injury and subsequent demand on tissue associated stem-cells, augmenting BMDC contribution. Nevertheless, the frequency of observed donor-derived muscle fibres and cardiomyocytes detected in this study were too few to achieve any functional restoration of dystrophin expression and subsequent improvement of phenotype. Of the fusion events observed, re-programming was indicated by onset of dystophin expression in GFP^+^ myocytes in skeletal muscle and myocardium.

It was hypothesized that the dystrophic phenotype would worsen with age and that the frequency of BMDC muscle reconstitution would increase accordingly, however no correlation between worsening phenotype and improved engraftment was observed. Fusion events in both skeletal muscle and myocardium were extraordinarily rare under the normal pathological conditions of muscular dystrophy in this model. In the absence of irradiation induced injury, only two GFP^+^ cardiomyocytes and fifteen GFP^+^ muscle fibres were detected 24 weeks post BMT. It was expected that the frequency of fusion in the hearts of W^41^Dmd^mdx^ mice would be similar to frequencies observed in infarcted mice [1]. However, our data indicate that the percentage frequency of fusion events between irradiated and non-irradiated tissues are similar in both skeletal muscle and heart, when compensated for the frequency of GFP^+^ blood cell reconstitution (**Table 1**).

It is clear from these results that in the absence of irradiation, the formation of stable and reprogrammed fusion hybrids occurs at extremely low or undetectable levels in non-irradiated recipients. The cardiac muscle degeneration associated with the dystrophic phenotype was inadequate pre-conditioning for the recruitment of BMDCs and failed to trigger physiological mechanisms such as local inflammation [10], mobilisation and homing of hematopoietic cells. It is likely that the relatively mild muscle degeneration in the Dmd*^mdx^* mouse model [18]–[19] in comparison with DMD patients contributes to the low frequency of BMDC engraftment observed. Also, the functional requirement of dystrophin to transmit muscle force is far less in mice than humans. Exercise induced destruction, correlative with irradiation damage, has been shown to enhance the contribution of GFP^+^ bone marrow derived cells to the myogenic phenotype in the Dmd*^mdx^* model [12]. Similarly, in studies of other tissues such as liver, irradiation was necessary for BMDC conversion to liver [20]–[21], but in conjunction with chemical or genetic damage-inducing discriminating pressures [3], [5], [7].

## Supporting Information

Figure S1 **Mononucleated GFP+ cells co-express CD45 and are confined predominantly to the interstitial cavities and vessels in un-irradiated tissue.** Myocardium of W^41^/Dmd*^mdx^* mouse 24 weeks post transplantation. Cardiomyocytes stained with c-Troponin-T (top left panel), and DAPI (top right panel) GFP and CD45 co-localization (bottom left and bottom right panel.). Scale bars represent 100 µm.
(TIF)
