# Supplementary figures and images for: Myogenic Reprogramming of Bone Marrow Derived Cells in a W41Dmdmdx Deficient Mouse Model
Source: PLoS One. 2011 Nov 28;6(11):e27500. doi: 10.1371/journal.pone.0027500 (PMC3225365; doi:10.1371/journal.pone.0027500)

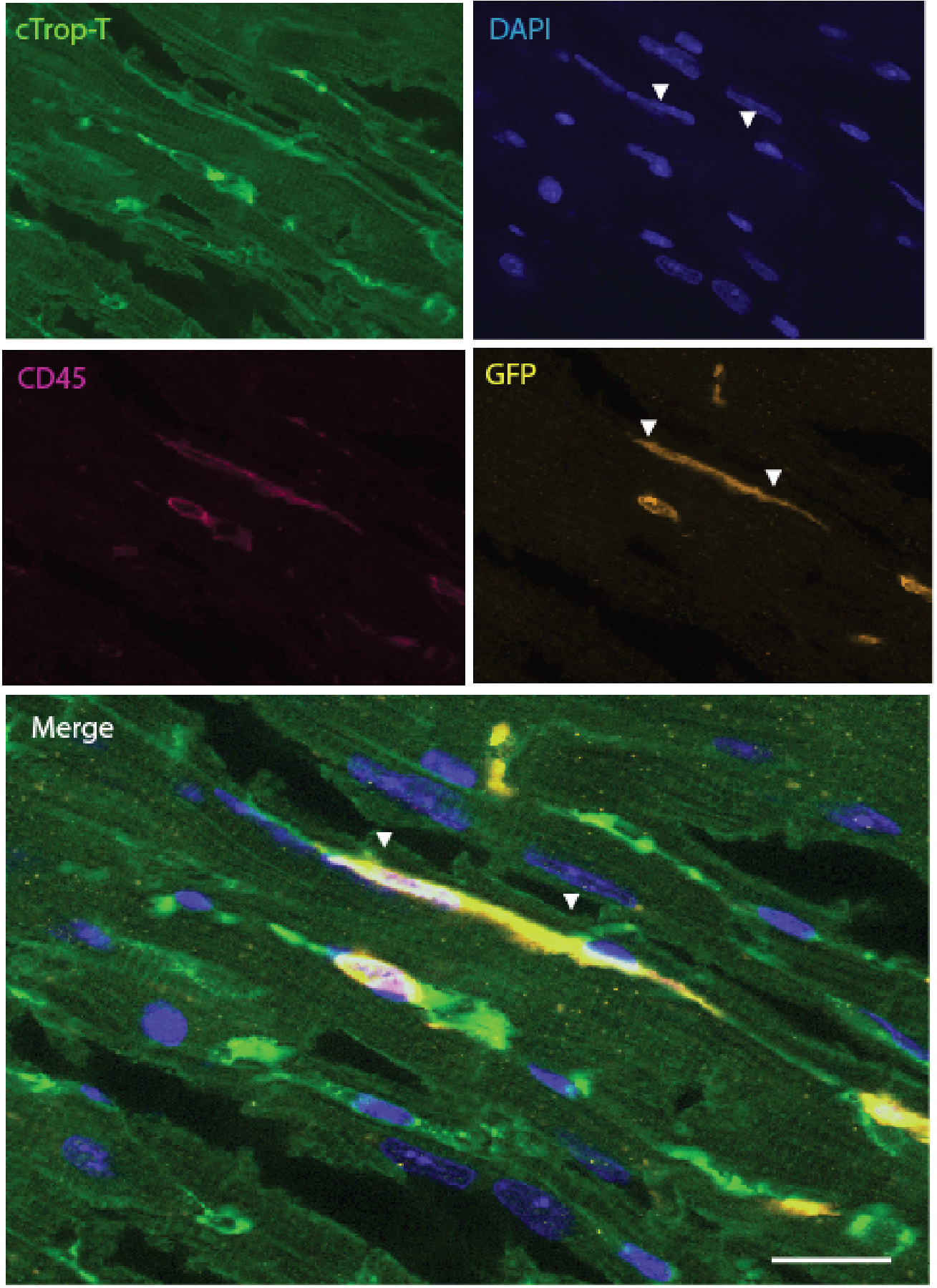

Supplement: Figure S1 — Mononucleated GFP+ cells co-express CD45 and are confined predominantly to the interstitial cavities and vessels in un-irradiated tissue. Myocardium of W41/Dmdmdx mouse 24 weeks post transplantation. Cardiomyocytes stained with c-Troponin-T (top left panel), and DAPI (top right panel) GFP and CD45 co-localization (bottom left and bottom right panel.). Scale bars represent 100 µm. (TIF) [file pone.0027500.s001.tif]
